# Blood Neutrophil Counts Define Specific Clusters of Bronchiectasis Patients: A Hint to Differential Clinical Phenotypes

**DOI:** 10.3390/biomedicines10051044

**Published:** 2022-04-30

**Authors:** Xuejie Wang, Casilda Olveira, Rosa Girón, Marta García-Clemente, Luis Máiz, Oriol Sibila, Rafael Golpe, Rosario Menéndez, Juan Rodríguez-López, Concepción Prados, Miguel Angel Martinez-García, Juan Luis Rodriguez, David de la Rosa, Liyun Qin, Xavier Duran, Jordi Garcia-Ojalvo, Esther Barreiro

**Affiliations:** 1 Lung Cancer and Muscle Research Group, Pulmonology Department, Hospital del Mar-IMIM, Parc de Salut Mar, 08003 Barcelona, Spain; xuejie.wang@e-campus.uab.cat (X.W.); liyun.qin@e-campus.uab.cat (L.Q.); 2 Department of Medicine, Universitat Autònoma de Barcelona (UAB), 08193 Barcelona, Spain; 3 Respiratory Department, Instituto de Investigación Biomédica de Málaga (IBIMA), Hospital Regional Universitario de Málaga, Universidad de Málaga, 29010 Málaga, Spain; casi1547@separ.es; 4 Respiratory Department, Instituto de Investigación Sanitaria, Hospital Universitario de la Princesa, 28006 Madrid, Spain; rosamaria.giron@salud.madrid.org; 5 Respiratory Department, Hospital Universitario Central de Asturias, 33011 Oviedo, Spain; marta.garciac@sespa.es; 6 Respiratory Department, Hospital Ramon y Cajal, 28034 Madrid, Spain; luis.maiz@salud.madrid.org; 7 Respiratory Department, Hospital Clínic, 08036 Barcelona, Spain; osibila@clinic.cat; 8 Centro de Investigación en Red de Enfermedades Respiratorias (CIBERES), Instituto de Salud Carlos III (ISCIII), 28220 Madrid, Spain; martinez_miggar@gva.es; 9 Respiratory Department, Hospital Lucus Augusti, 27003 Lugo, Spain; rafa898@separ.es; 10 Respiratory Department, Hospital Universitario y Politécnico La Fe, 46026 Valencia, Spain; menendez_ros@gva.es; 11 Respiratory Department, Hospital San Agustin, 33401 Avilés, Spain; juan.rodriguez@sespa.es; 12 Respiratory Department, Hospital Universitario la Paz, 28046 Madrid, Spain; mconcepcion.prados@salud.madrid.org; 13 Respiratory Department, Hospital Clínico San Carlos, 28040 Madrid, Spain; jlrodr01@ucm.es; 14 Instituto de Investigación Sanitaria del Hospital Clínico San Carlos (IdISSC), 28040 Madrid, Spain; 15 Departamento de Medicina, Universidad Complutense de Madrid, 28040 Madrid, Spain; 16 Respiratory Department, Hospital Santa Creu I Sant Pau, 08025 Barcelona, Spain; drosa@santpau.cat; 17 Scientific and Technical Department, Hospital del Mar (IMIM), 08003 Barcelona, Spain; xduran@imim.es; 18 Department of Medicine and Life Sciences (MELIS), Universitat Pompeu Fabra (UPF), 08002 Barcelona, Spain; jordi.g.ojalvo@upf.edu

**Keywords:** neutrophilic inflammation, bronchiectasis, biostatistics analyses, phenotypic clusters, clinical outcomes, bronchiectasis severity scores, multivariate analyses

## Abstract

We sought to investigate differential phenotypic characteristics according to neutrophil counts, using a biostatistics approach in a large-cohort study from the Spanish Online Bronchiectasis Registry (RIBRON). The 1034 patients who met the inclusion criteria were clustered into two groups on the basis of their blood neutrophil levels. Using the Mann–Whitney U test to explore potential differences according to FACED and EFACED scores between the two groups, a neutrophil count of 4990 cells/µL yielded the most balanced cluster sizes: (1) above-threshold (*n* = 337) and (2) below-threshold (*n* = 697) groups. Patients above the threshold showed significantly worse lung function parameters and nutritional status, while systemic inflammation levels were higher than in the below-threshold patients. In the latter group, the proportions of patients with mild disease were greater, while a more severe disease was present in the above-threshold patients. According to the blood neutrophil counts using biostatistics analyses, two distinct clinical phenotypes of stable patients with non-CF bronchiectasis were defined. Patients falling into the above-threshold cluster were more severe. Severity was characterized by a significantly impaired lung function parameters and nutritional status, and greater systemic inflammation. Phenotypic profiles of bronchiectasis patients are well defined as a result of the cluster analysis of combined systemic and respiratory variables.

## 1. Introduction

Neutrophilic inflammation is a hallmark in the pathophysiology of several chronic respiratory diseases, such as chronic obstructive pulmonary disease (COPD), cystic fibrosis, and bronchiectasis [1]. Neutrophils are key cells of the immune system that possess a strong destructive capacity as a result of their powerful toxins. These cells were shown to play a crucial role in the pathogenesis of chronic airway diseases [1].

Bronchiectasis is characterized by the dilatation of the airways along with impaired mucociliary function. Patients usually experience cough and increased mucus production with frequent exacerbations due to bacterial infection [2]. Chronic bronchial and lung infection is common in patients with some chronic inflammatory lung diseases, including bronchiectasis [3,4,5,6]. Persistent microbial dysbiosis leads to neutrophilic inflammation leading to tissue damage as a result of the release of serine proteases in these patients [7,8]. Moreover, defects in the function of neutrophils have also been previously demonstrated in bronchiectasis [9,10,11]. As such, bacterial phagocytosis and destruction [9], increased levels of necrotic neutrophils and altered inflammation resolution [10], spontaneous neutrophil apoptosis, and increased formation of neutrophil extracellular traps (NET) [11] have been described in different investigations. Whether the defective neutrophil phenotype is the result of bronchiectasis or the consequence of the persistent inflammatory milieu in the airways of these patients remains to be fully elucidated.

Biostatistical tools, which combine the use of specific software with large patient and/or biological data sets, may help identify specific phenotypes with a potential clinical applicability. The phenotypic characterization of patients with bronchiectasis is needed in order to establish a more personalized therapeutic approach. Specific phenotypes have been defined on the basis of the eosinophil counts in a large cohort of patients with bronchiectasis [12]. In another investigation [13], several blood parameters allowed for the identification of three different clinical phenotypes in bronchiectasis patients in another large cohort of patients. In both investigations, disease severity, lung function, and systemic inflammatory and nutritional parameters clearly differed among the clusters of patients obtained from the biostatistical approach used in each type of investigation [12,13].

Hence, in the current investigation, we hypothesized whether specific clinical phenotypes can be defined on the basis of blood neutrophil counts in a large cohort of bronchiectasis patients from the Spanish Online Bronchiectasis Registry (RIBRON). Accordingly, the study objectives were defined as follows: (1) to tease out a cut-off value of blood neutrophil counts that may discriminate differential phenotypic clusters of patients, (2) to examine clinical differences (disease severity scores, smoking history, lung function, and systemic inflammatory and nutritional status), and (3) to stratify the clusters according to well-known bronchiectasis severity scores to explore the potential associations with blood neutrophil counts.

## 2. Materials and Methods

### 2.1. Study Design

The current investigation was conducted within the frame of RIBRON study (43 centers from Spain), in which patients were recruited from February 2015 and October 2019. The study design was observational and prospective. Strengthening the Reporting of Observational Studies in Epidemiology (STROBE) guidelines were used [14]. An external contract research organization (CRO) monitored the quality of the data introduced in the registry at all times.

### 2.2. Study Population

**Figure 1:**
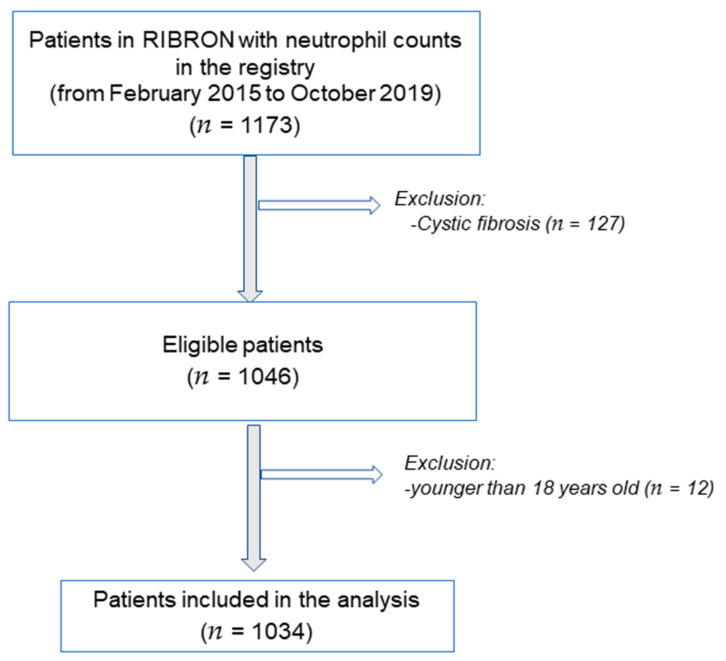
Schematic representation of the flow of the study.

Figure 1 illustrates the flowchart of the investigation. Inclusion criteria were as follows: adult patients who had been diagnosed with non-CF bronchiectasis as a result of high-resolution computerized tomography (HRCT) [4,5,15,16,17,18]. A total of 1034 patients were analyzed from the registry in the present study. General clinical data, such as anthropometry, smoking history, lung function, hemogram, inflammatory blood cells, and nutritional parameters, were analyzed using custom data-analysis software tools. At the time of study entry, all the patients were stable and had not had any acute exacerbation at least in the last four weeks prior to study entry. Exclusion criteria were established as follows: age younger than 18 years old, traction bronchiectasis and/or cystic fibrosis. The research followed the guidelines of the World Medical Association for Research in Humans (seventh revision of the Declaration of Helsinki, Fortaleza, Brazil, 2013) [19]. Ethics approval was obtained from the Ethics Committee at the Hospital Josep Trueta Girona (# 001-2012, Hospital Universitari Dr. Josep Trueta, Girona, Spain) in the coordinating center and in the local participating centers. All the participants signed the informed written consent prior to study entry. The information remained confidential at all times. Personal data were never introduced in the registry.

### 2.3. Clinical Variables and Scores

Clinical variables and analytical parameters were obtained from the study patients: anthropometry (age and body mass index), lung function, chronic colonization by *Pseudomonas aeruginosa* (PA), radiologic extension, dyspnea, the number of exacerbations and hospitalizations for exacerbations in the previous year, the Charlson index, smoking history, nutritional status, and systemic inflammatory cells and markers. Disease severity scores were also calculated for all the patients as follows: FACED (FEV_1_, age, chronic colonization by PA, radiological extension, and dyspnea) [20], EFACED (exacerbation FACED) [21], and BSI (bronchiectasis severity index) [22].

### 2.4. Clusters of Patients

A threshold of neutrophils was established on the basis of the FACED and EFACED scores for all the study population. The highest threshold of neutrophil counts was established as 4990 cells/µL. This cut-off value evidenced a statistically significant difference in FACED and EFACED scores between the two groups of patients, while maintaining a relative balance in the size of each patient group.

### 2.5. Statistical Analysis

Differences for all the study variables were assessed between the two study groups: above- and below-threshold patients using the T-student test for the quantitative variables and the Chi-square test for the categorical variables. In the tables, the study variables are presented as means and standard deviations. In figures, the distribution of dichotomized neutrophils (above versus below threshold) for each disease severity score (EFACED, BSI and FACED) are shown in histograms. Patients with concomitant COPD were excluded from the analysis in order to check the potential influence of this disease on the study results.

Potential correlations between two study variables were assessed, using the Pearson’s correlation coefficient. Additionally, graphical correlation matrixes were depicted using the R package corrplot (https://cran.r-project.org/web/packages/corrplot/index.html, accessed on 15 February 2022). Blue dots indicated the existence of a positive correlation between two variables, while the red dots represented the negative correlations.

Disease severity scores defined as FACED ≥ 5 or lower than 5 and EFACED ≥ 7 or lower than 7 were defined to assess associations of dichotomized neutrophils using multivariate logistic regression analysis. Potential clinically meaningful confounders were established as follows: chronic colonization by PA, Charlson index, the total number of leukocytes, the percentage of lymphocytes and eosinophils, the number of platelets, and C-reactive protein (CRP), fibrinogen, protein and albumin levels, and erythrocyte sedimentation rate (ESR). Statistical analyses were performed using Stata 15.1 (StataCorp LLC, College Station, TX, USA). Statistical significance was established at *p* < 0.05 for all the tests.

## 3. Results

### 3.1. Identification of the Best Cut-Off Value of Neutrophils

**Figure 2:**
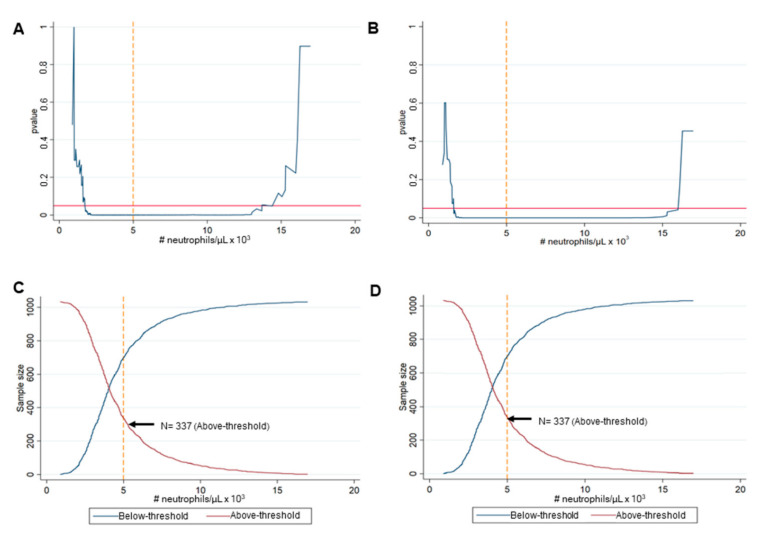
Identification of the neutrophil threshold for patient clustering. Classification of neutrophil counts based on (**A**) FACED and (**B**) EFACED: Statistical significance resulting from the Mann–Whitney U test as a measure of the corresponding p-value for varying thresholds The statistical significance threshold *p* = 0.05 is represented in the horizontal solid red line, while the vertical yellow dashed line represents the optimal threshold of neutrophil counts Number of patients in the above-threshold cluster based on (**C**) FACED and (**D**) EFACED. Black arrows point toward the percentage of the patients’ threshold in which the most balanced cluster sizes were attained.

The threshold of neutrophil counts was 4900 cells/µL for both FACED and EFACED scores as indicated in Figure 2A,B. Statistically significant differences between the above- and below-threshold clusters of patients were detected with a concentration of neutrophils of 4900 cells/µL for the FACED and EFACED scores (*p* < 0.001, Figure 2A,B). Thirty-two percent of the patients (337 out of 1034) were in the above-threshold group. Interestingly, the most balanced patient clusters were achieved with this threshold of neutrophil concentration. Statistically significant differences in FACED and EFACED were equally observed between the two patient clusters with this threshold of neutrophil levels (Figure 2C,D, respectively).

### 3.2. Clinical Characteristics

Age was significantly greater in the above-threshold than in the below-threshold patients (Table 1). Total body mass index did not differ between the two patient clusters. Similar results were observed when COPD patients (*n* = 119) or patients with chronic colonization by PA (*n* = 263) were excluded from this analysis (Table 2 and Table 3, respectively).

**Figure 3:**
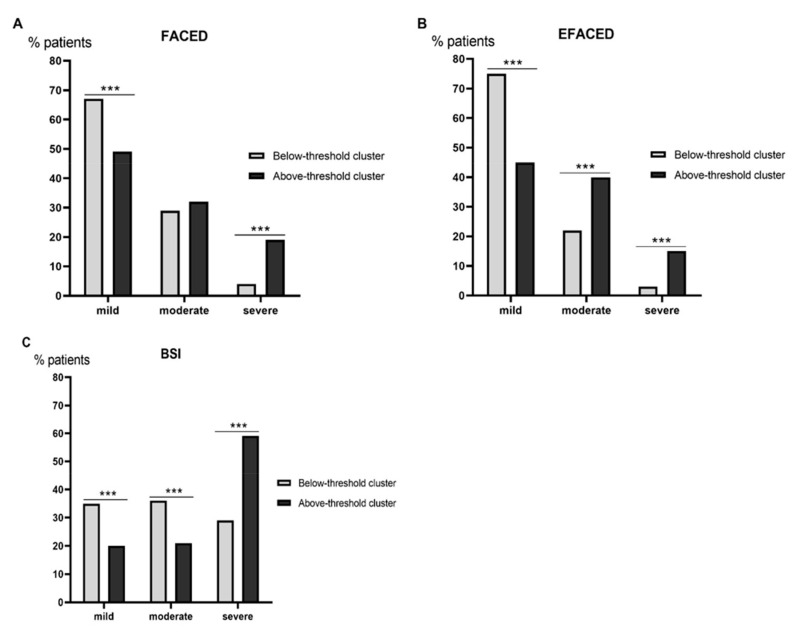
Histograms of the proportions of patients who were classified as mild–moderate–severe according to FACED (**A**), EFACED (**B**), and BSI (**C**) between the two patient clusters. Disease severity scores were calculated as follows: FACED: mild: 0–2, moderate: 3 to 4, and severe: 5–7; EFACED: mild: 0–3, moderate: 4–6, and severe: 7–9; and BSI: mild: 0–4, moderate: 5–8, and severe: ≥9. Statistical significance: *** *p* < 0.001 between the two patient groups.

Scores of disease severity (FACED, EFACED, and BSI), the number of exacerbations and hospitalizations, the Charlson index, and the proportion of patients with chronic colonization by PA and COPD were greater in the above-threshold cluster than in the below-patient group (Table 1). The proportion of patients with asthma did not differ between the two patient groups (Table 1). Similar results were encountered when patients with concomitant COPD or chronic colonization by PA were excluded from the analysis (Table 2 and Table 3, respectively). A greater proportion of below-threshold patients fell into the mild category for all scores (FACED, EFACED, and BSI, Figure 3A–C, respectively). Conversely, a larger proportion of above-threshold patients fell into the severe category for all three scores (FACED, EFACED, and BSI, Figure 3A–C, respectively). Nonetheless, discrepancies were observed for the moderate category, in which a greater proportion of above-threshold patients was observed for the EFACED score, while for the BSI score, a larger proportion of below-threshold patients was detected (Figure 3A–C, respectively).

Smoking history was more prominent in patients of the above-threshold cluster (Table 1), and this was not related to COPD or chronic colonization by PA (Table 2 and Table 3, respectively). Patients in the above-threshold showed a greater degree of airway obstruction and worse diffusion capacity than patients in the below-threshold group, and those were independent of the presence of COPD or chronic colonization by PA (Table 1, Table 2, Table 3, respectively). The presence of comorbidities for the two clusters of patients is shown in Table 4. Briefly, above-threshold patients exhibited greater proportions of myocardial infarction, heart and kidney failure, dementia, and diabetes than below-threshold patients (Table 4).

### 3.3. Profile of the Systemic Parameters in the Two Patient Clusters

As expected, levels of total leukocytes and neutrophils were higher in the above-threshold cluster than in the below-threshold patients, and these results were not influenced by the presence of COPD or chronic colonization by PA (Table 5, Table 6 and Table 7, respectively). Eosinophil counts were significantly reduced in the above-threshold cluster compared to the below-threshold group of patients, and similar findings were shown when COPD patients or patients with chronic colonization by PA were excluded from the analysis (Table 5, Table 6 and Table 7). The number of platelets was significantly increased in the above-threshold group than in the below-threshold cluster, and this was independent of COPD or chronic colonization by PA (Table 5, Table 6 and Table 7, respectively). Importantly, levels of CRP, ESR, and fibrinogen were significantly greater in the above-threshold group than in the below-threshold patients, and these results were similar when patients with concomitant COPD or chronic colonization by PA were excluded from the analysis (Table 5, Table 6 and Table 7, respectively). Protein and albumin concentrations were significantly decreased in the above-threshold patients than in the below-threshold cluster, with similar results when COPD or chronic colonization by PA were excluded (Table 5, Table 6 and Table 7, respectively).

**Figure 4:**
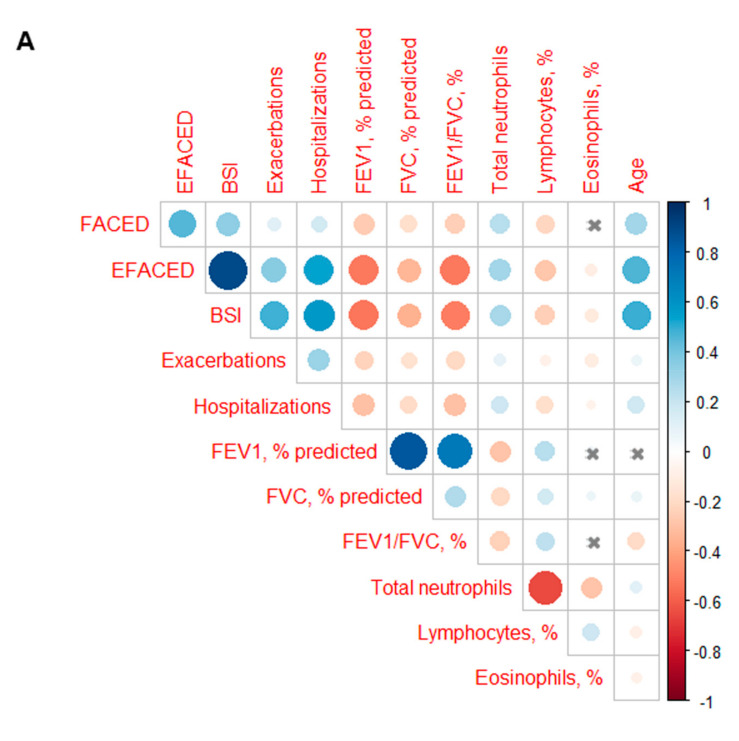

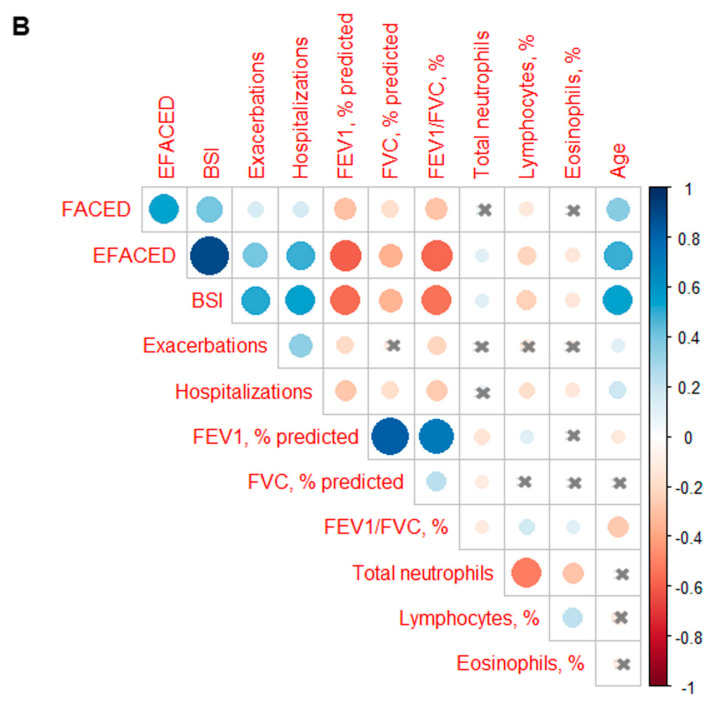

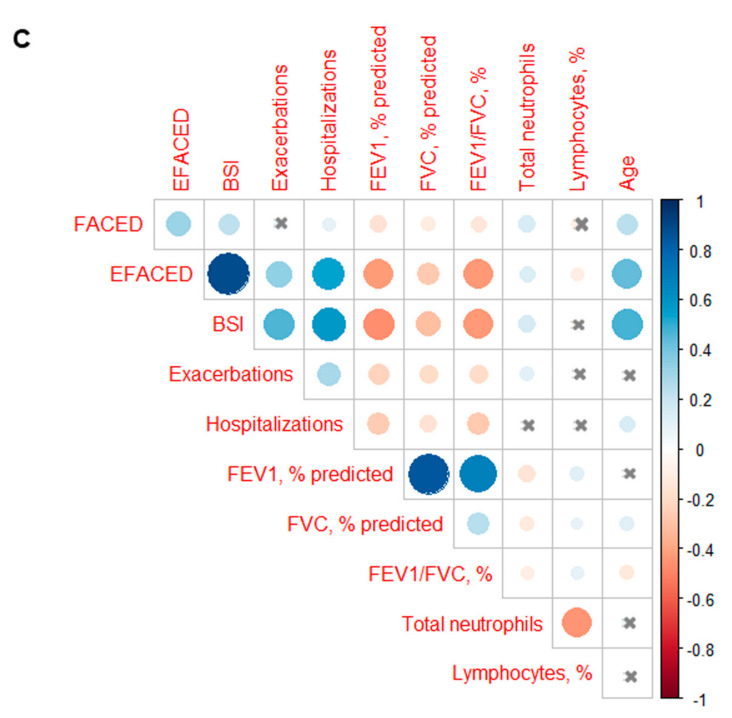
Correlation matrix of the disease severity and analytical variables in which blue dots indicate the existence of a positive correlation between two variables, while the red dots represent the existence of negative correlations: (**A**) all the study patients, (**B**) patients in the above-threshold cluster, (**C**) patients in the below-threshold cluster. A *p* value > 0.05 is represented as the intersection within the circle. The color intensity and the size of the circle are proportional to the correlation coefficients, as indicated in the Y-axis on the right-hand side of the graph.

Among all the patients as a whole, significant positive correlations were detected between neutrophil counts and the scores FACED, EFACED, and BSI, the number of exacerbations and hospitalizations (Figure 4A). Neutrophil counts negatively correlated with the lung function parameters FEV_1_ and FEV_1_/FVC when patients were analyzed altogether (Figure 4A). In the above-threshold cluster, weak significant positive correlations were also observed between the neutrophil counts and the scores EFACED and BSI, but not with the number of exacerbations or hospitalizations (Figure 4B). In the below-threshold cluster of patients, significant positive correlations were observed between neutrophil counts and all three scores (FACED, EFACED, and BSI) and the number of exacerbations (Figure 4C). Very weak negative correlations were also detected between neutrophil levels and the lung function variables FEV_1_, FVC, and FEV_1_/FVC among the below-threshold cluster of patients (Figure 4C).

### 3.4. Multivariate Analyses

**Figure 5:**
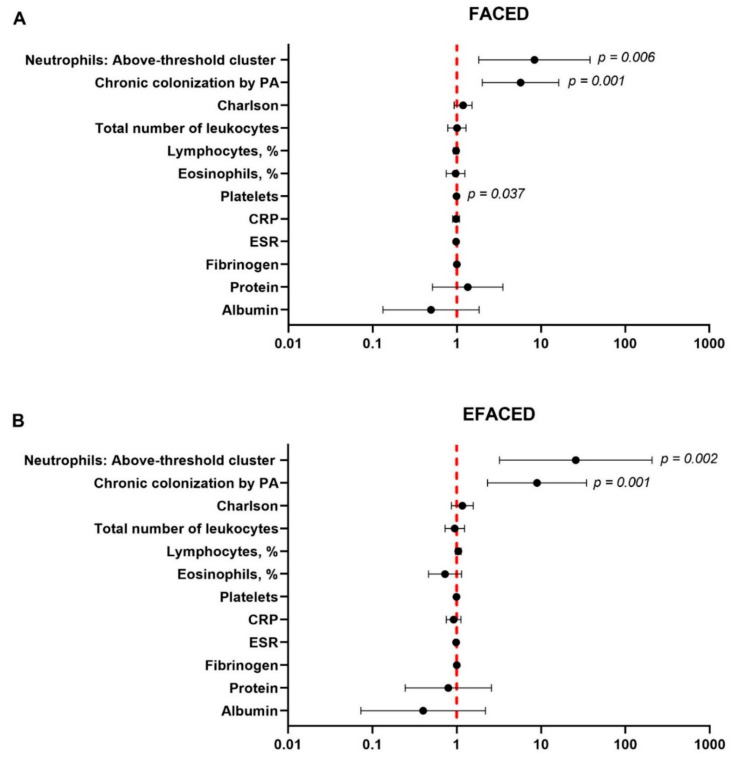
The independent association of dichotomized neutrophils with the values of (**A**) FACED and (**B**) EFACED. A multivariate regression analysis was used to perform the analysis. The confidence intervals and statistical significance are represented in the figure panel. The odds ratio is represented as black dots. The model was adjusted by the chronic colonization by *Pseudomonas aeruginosa*, Charlson index, the total number of leukocytes, the percentage of lymphocytes and eosinophils, the number of platelets, and C-reactive protein (CRP), fibrinogen, protein and albumin levels, and erythrocyte sedimentation rate (ESR).

Dichotomized neutrophil levels were significantly associated with greater values of FACED and EFACED scores (odds ratio (OR) = 8.37; CI95%: 1.82–38.39, *p* value = 0.006 and OR = 25.84; CI95%: 3.22–207.24, *p* = 0.002, respectively) as well as with the proportions of chronic colonization by PA (Figure 5A and Figure 5B, respectively). This analysis further confirms the approach of the identification of the two patient clusters according to the neutrophil concentrations. Patients of the above-threshold group were those exhibiting a higher risk to attain greater FACED and EFACED scores (OR > 1), as demonstrated by the multivariate model after adjusting for potential confounders (Figure 5A,B).

## 4. Discussion

Two different phenotypes were identified according to the number of neutrophils in this study, as a result of a biostatistics analysis of a large cohort of bronchiectasis patients. A cut-off value of 4990 neutrophils/µL was defined on the basis of a statistical approach that enabled us to categorize the patients into two groups with a clear differential phenotype. While patients in the above-threshold cluster exhibited a more severe disease and a worse lung function along with a significant rise in systemic inflammatory parameters, those who fell into the below-threshold cluster showed a less severe disease for all the target variables. A discussion of the most relevant findings follows below.

Patients who fell into the above-threshold phenotype were those exhibiting greater values of the disease severity scores as indicated in the tables. Importantly, the proportions of patients with a more severe disease according to the three study scores (FACED, EFACED, and BSI) were those of the above-threshold cluster. Additionally, significant inverse correlations, although weak, were also detected between neutrophil levels and spirometric parameters in the above-threshold cluster of patients. In fact, when patients were analyzed as a whole, significant inverse correlations were also detected between the neutrophil counts and the same spirometric variables. These results suggest that systemic neutrophil levels are directly correlated with the degree of airway obstruction. These observations are in line with the predominant neutrophilic phenotype observed in patients with obstructive lung diseases, namely bronchiectasis and COPD [1,23,24,25].

Importantly, the number of exacerbations and hospitalizations positively correlated with the neutrophil counts when the entire population of patients was analyzed. These correlations were not found when the above-threshold patients were studied independently, probably due to the lower number of the target patients (*n* = 337). Similarly, recent observations demonstrated that high numbers of blood neutrophils also helped predict the risk of exacerbations and mortality among patients with COPD [26]. Likewise, neutrophil dysfunction was also recently proposed as a target mechanism for the design of novel therapeutic strategies in bronchiectasis [27].

In the above-threshold cluster of patients, the numbers of total leukocytes and neutrophils were significantly increased, compared to patients classified in the below-threshold cluster of patients. In a way, these results were expected findings since the level of neutrophils was the target variable used to define the two different phenotypic clusters in the study. Similarly, platelet counts were also greater in the above-threshold group of patients than in the below-threshold cluster. Furthermore, levels of systemic inflammatory parameters, such as CRP, ESR, and fibrinogen, were significantly higher in the above-threshold patients. These are important findings that reveal that patients with a rise in neutrophil counts are those showing greater levels of systemic inflammation. Despite the current study not being longitudinal, it would be possible to conclude that increased systemic inflammation may worsen disease progression and morbidity in patients with bronchiectasis, as previously demonstrated in a large cohort follow-up study [28].

The number of eosinophils was significantly reduced in the above-threshold cluster. These findings are in agreement with a previous investigation [12], in which bronchiectasis patients with a level of eosinophils lower than 100 cells/µL were those exhibiting a more severe disease as measured by EFACED and BSI and worse lung function parameters. It is possible to conclude that low numbers of systemic eosinophils and high neutrophil counts are relevant contributors to disease severity, progression in bronchiectasis and response to treatment [29]. Future investigations should elucidate the specific role of these cell types in the course of the disease.

Total protein and albumin levels, although within the normal range in both groups of patients, were significantly reduced in the above-threshold cluster of patients. In patients with low levels of eosinophils, protein and albumin systemic levels were also diminished, compared to patients with eosinophil levels greater than 100 cells/µL [12]. Whether increased levels of neutrophils along with reduced counts of eosinophils may be associated with a poorer nutritional status remains to be assessed in future investigations. However, a previous study put the line forward that high levels of eosinophils were associated with a better nutritional status and improved lung function in patients with emphysema [30].

### Study Critique

A limitation in the study is related to its descriptive nature. Several relevant questions have arisen from the current investigation. Elucidation of the potential biological mechanisms underlying the clinical phenotypes will require the design of specific investigations aimed to identify the underlying biology. Moreover, follow-up studies will have to be designed with the aim to explore whether the findings reported herein have a prognostic value, namely the neutrophil counts in each specific cluster of patients. Future investigations should also focus on an external validation of the reported findings in a different study cohort of bronchiectasis patients, in which a large number of patients are to be analyzed using a multicenter approach.

## 5. Conclusions

Two distinct clinical phenotypes of stable patients with bronchiectasis of a wide range of disease severity were established on the basis of blood neutrophil counts, using a biostatistics approach. Patients classified within the above-threshold cluster were those exhibiting a severe disease, significantly worse clinical outcomes, lung function parameters and nutritional status, while showing greater levels of systemic inflammatory parameters. These results will contribute to better characterize bronchiectasis patients into phenotypic profiles with their clinical implications.

## Figures and Tables

**Table 1:** General characteristics of all the study patients according to the two clusters of patients.

	Below-Threshold Cluster	Above-Threshold Cluster
	<4.99 × 10^3^ Cells/µL	≥4.99 × 10^3^ Cells/µL
	(N = 697)	(N = 337)
**Anthropometric variables,** $\bar{x}\;$(SD)		
Age, years	67.1 (14.6)	70.3 (15.6) **
BMI, kg/m^2^	25.7 (4.9)	26.2 (5.2)
**Disease severity,** $\bar{x}\;$(SD)		
FACED score	1.9 (1.5)	2.8 (1.9) ***
EFACED score	2.4 (1.9)	3.8 (2.4) ***
BSI	6.9 (4.3)	9.6 (5.1) ***
Exacerbations	1.5 (1.6)	1.7 (1.6) *
Hospitalization	0.5 (1.2)	1.1 (1.7) ***
Charlson Index	1.8 (1.4)	2.2 (1.9) ***
Radiological extension	2.88 (1.41)	2.83 (1.39)
Chronic colonization by PA, N (%)	150 (21.5)	113 (33.5) ***
Asthma, N (%)	70 (10)	36 (10.7)
COPD, N (%)	57 (8.2)	62 (18.4) ***
**Smoking history**		
Never smokers, N (%)	443 (64)	162 (48) ***
Current smokers, N (%)	51 (7)	40 (12) *
Ex-smokers, N (%)	203 (29)	135 (40) **
Packs-year, $\bar{x}\;$(SD)	28.2 (24.8)	35.4 (27.2) **
**Lung function,**$\bar{x}\;$(SD)		
FEV_1_, % predicted	78 (24)	64 (25) ***
FVC, % predicted	88 (21)	79 (23) ***
FEV_1_/FVC, %	70 (12)	64 (14) ***
DL_CO_, % predicted	86 (22)	73 (25) ***
K_CO_, % predicted	80 (36)	69 (41) *
RV, % predicted	137 (45)	147 (58)
TLC, % predicted	102 (18)	102 (24)
RV/TLC, %	50 (11)	52 (13)

Continuous variables are presented as mean (standard deviation), while categorical variables are presented as the number of patients in each group along with the percentage for the study group. Definition of abbreviations: N, number; $\bar{x}\;$, mean; SD, standard deviation; m, meters; kg, kilograms; BMI, body mass index; FACED: F, FEV1; A, Age; C, chronic colonization by *Pseudomonas aeruginosa*; E, radiologic extension; D, dyspnea; EFACED: exacerbation FACED; BSI, bronchiectasis severity index; PA, *Pseudomonas aeruginosa*; FEV_1_, forced expiratory volume in the first second; FVC, forced vital capacity; RV, residual volume; TLC, total lung capacity; DLco, carbon monoxide transfer; K_CO_, Krogh transfer factor. Statistical analyses and significance: * *p* < 0.05; ** *p* < 0.01; *** *p* < 0.001 between the patient groups.

**Table 2:** General characteristics in the two clusters of bronchiectasis patients, excluding those with COPD.

	Below-Threshold Cluster	Above-Threshold Cluster
	<4.99 × 10^3^ Cells/µL	≥4.99 × 10^3^ Cells/µL
	(N = 640)	(N = 275)
**Anthropometric variables,** $\bar{x}\;$(SD)		
Age, years	66.4 (14.7)	68.6 (16.2) *
BMI, kg/m^2^	25.6 (4.9)	26.1 (5.3)
**Disease severity,** $\bar{x}\;$(SD)		
FACED score	1.8 (1.5)	2.5 (1.8) ***
EFACED score	2.3 (1.9)	3.4 (2.3) ***
BSI	6.7 (4.2)	8.8 (4.8) ***
Exacerbations	1.5 (1.6)	1.6 (1.5)
Hospitalization	0.5 (1.2)	1 (1.6) ***
Charlson Index	1.7 (1.3)	2.1 (2) **
Radiological extension	2.9 (1.4)	2.8 (1.4)
Chronic colonization by PA, N (%)	143 (22.3)	90 (32.7) **
**Smoking history**		
Never smokers, N (%)	438 (68)	161 (59) **
Current smokers, N (%)	38 (6)	30 (11) *
Ex-smokers, N (%)	164 (26)	84 (30)
Packs-year, $\bar{x}\;$(SD)	22.3 (19.7)	28.4 (23.3) *
**Lung function,**$\bar{x}\;$(SD)		
FEV_1_, % predicted	80 (23)	68 (25) ***
FVC, % predicted	88 (21)	81 (24) ***
FEV_1_/FVC, %	71 (11)	67 (13) ***
DL_CO_, % predicted	87 (22)	78 (23) **
K_CO_, % predicted	80 (37)	72 (39)
RV, % predicted	133 (43)	143 (55)
TLC, % predicted	101 (18)	99 (22)
RV/TLC, %	49 (11)	52 (13)

Continuous variables are presented as mean (standard deviation), while categorical variables are presented as the number of patients in each group along with the percentage for the study group. Definition of abbreviations: N, number; $\bar{x}\;$, mean; SD, standard deviation; m, meters; kg, kilograms; BMI, body mass index; FACED: F, FEV_1_; A, age; C, chronic colonization by *Pseudomonas aeruginosa*; E, radiologic extension; D, dyspnea; EFACED: exacerbation FACED; BSI, bronchiectasis severity index; PA, *Pseudomonas aeruginosa*; FEV_1_, forced expiratory volume in the first second; FVC, forced vital capacity; RV, residual volume; TLC, total lung capacity; DLco, carbon monoxide transfer; K_CO_, Krogh transfer factor. Statistical analyses and significance: * *p* < 0.05; ** *p* < 0.01; *** *p* < 0.001 between the patient groups.

**Table 3:** General characteristics in the two clusters of bronchiectasis patients, excluding those with chronic colonization by PA.

	Below-Threshold Cluster	Above-Threshold Cluster
	<4.99 × 10^3^ Cells/µL	≥4.99 × 10^3^ Cells/µL
	(N = 547)	(N = 224)
**Anthropometric variables,** $\bar{x}\;$(SD)		
Age, years	67.4 (14.1)	69.6 (15.3) *
BMI, kg/m^2^	25.9 (4.9)	26.2 (5.0)
**Disease severity,** $\bar{x}\;$(SD)		
FACED score	1.5 (1.3)	2.2 (1.7) ***
EFACED score	2 (1.7)	3.1 (2.2) ***
BSI	5.8 (3.6)	7.9 (4.4) ***
Exacerbations	1.3 (1.6)	1.4 (1.5)
Hospitalization	0.4 (1)	0.9 (1.3) ***
Charlson Index	1.8 (1.4)	2 (1.9), *p* = 0.065
Radiological extension	2.76 (1.38)	2.67 (1.43)
**Smoking history**		
Never smokers, N (%)	323 (59)	97 (43) ***
Current smokers, N (%)	47 (9)	35 (16) **
Ex-smokers, N (%)	177 (32)	92 (41) *
Packs-year, $\bar{x}\;$(SD)	27.8 (24.3)	33.2 (22.1) *
**Lung function,** $\bar{x}\;$(SD)		
FEV_1_, % predicted	82 (23)	68 (25) ***
FVC, % predicted	90 (20)	82 (23) ***
FEV_1_/FVC, %	71 (11)	65 (15) ***
DL_CO_, % predicted	87 (22)	74 (27) ***
K_CO_, % predicted	79 (37)	67 (41) *
RV, % predicted	135 (44)	145 (55)
TLC, % predicted	103 (17)	102 (24)
RV/TLC, %	49 (11)	52 (13)

Continuous variables are presented as mean (standard deviation), while categorical variables are presented as the number of patients in each group along with the percentage for the study group. Definition of abbreviations: N, number; $\bar{x}\;$, mean; SD, standard deviation; m, meters; kg, kilograms; BMI, body mass index; FACED: F, FEV_1_; A, Age; C, chronic colonization by *Pseudomonas aeruginosa*; E, radiologic extension; D, dyspnea; EFACED: exacerbation FACED; BSI, bronchiectasis severity index; PA, *Pseudomonas aeruginosa*; FEV_1_, forced expiratory volume in the first second; FVC, forced vital capacity; RV, residual volume; TLC, total lung capacity; DLco, carbon monoxide transfer; K_CO_, Krogh transfer factor. Statistical analyses and significance: * *p* < 0.05; ** *p* < 0.01; *** *p* < 0.001 between the patient groups.

**Table 4:** Comorbidities in the two clusters of patients.

Comorbidities, N (%)	Below-Threshold Cluster	Above-Threshold Cluster
Myocardial infarction	22 (3.2%)	20 (5.9%) *
Heart failure	42 (6%)	44 (13.1%) ***
Peripheral arterial disease	16 (2.3%)	15 (4.5%)
Cerebrovascular disease	24 (3.4%)	20 (5.9%)
Dementia	3 (0.4%)	6 (1.8%) *
Connective tissue disease	39 (5.6%)	24 (7.1%)
Gastroduodenal ulcer	53 (7.6%)	15 (4.5%)
Mild chronic liver disease	27 (3.9%)	11 (3.3%)
Diabetes	71 (10.2%)	51 (15.1) *
Diabetes with target organ damage	8 (1.1%)	12 (3.6%) *
Hemiplegia	0	2 (0.6%)
Moderate– severe chronic kidney failure	16 (2.3%)	20 (5.9%) **
Solid tumor or neoplasm	44 (6.3%)	30 (8.9%)
Leukemia	13 (1.9%)	6 (1.8%)
Lymphoma	9 (1.3%)	0
Moderate–severe chronic liver disease	9 (1.3%)	3 (0.9%)
Solid tumor or neoplasm with metastasis	6 (0.9%)	3 (0.9%)
Definite AIDS	1 (0.1%)	3 (0.9%)

Absolute number of patients and percentage for each comorbidity. Definition of abbreviations: N, number; %, percentage. Statistical significance: * *p* ≤ 0.05; ** *p* ≤ 0.01; *** *p* ≤ 0.001; between the two groups of patients.

**Table 5:** Inflammatory and nutritional parameters in the two clusters of patients.

	Below-Threshold Cluster	Above-Threshold Cluster
	<4.99 × 10^3^ Cells/µL	≥4.99 × 10^3^ Cells/µL
	(N = 697)	(N = 337)
**Blood parameters,** $\bar{x}\;$(SD)		
Total leukocytes, cells/µL	6.28 (2.61) × 10^3^	10.62 (4.16) × 10^3^ ***
Total neutrophils, cells/µL	3.37 (0.91) × 10^3^	7.53 (2.58) × 10^3^ ***
Neutrophils, %	54.79 (9.61)	71.01 (10.98) ***
Total lymphocytes, cells/µL	2.13 (2.27) × 10^3^	2.07 (3.15) × 10^3^
Lymphocytes, %	32.58 (9.43)	18.92 (9.03) ***
Total eosinophils, cells/µL	0.21 (0.20) × 10^3^	0.18 (0.19) × 10^3^
Eosinophils, %	3.27 (2.8)	1.8 (1.8) ***
Platelets, cells/µL	239 (67) × 10^3^	277 (85) × 10^3^ ***
Alpha-1 antitrypsin, mg/dL	133.23 (35.73)	137.62 (42.09)
CRP, mg/dL	2.89 (8.54)	5.31 (10.44) ***
ESR, mm/h	16.03 (14.62)	22.27 (19.98) ***
Fibrinogen, mg/dL	396.5 (109.5)	500.5 (162.9) ***
Hemoglobin, g/dL	13.61 (1.46)	13.5 (1.74)
Hematocrit, %	41.39 (4.12)	41.22 (5.02)
Creatinine, mg/dL	0.81 (0.43)	0.87 (0.46)
Total proteins, g/dL	7.04 (0.59)	6.89 (0.7) **
Albumin, g/dL	4.23 (0.41)	4.08 (0.51) ***

Continuous variables are presented as mean (standard deviation) for the study group. Definition of abbreviations: N, number; CRP, C-reactive protein; ESR, erythrocyte sedimentation rate; uL, microliter; dL, deciliter; mg, milligrams; mm, millimeters; h, hour; g, grams. Statistical analyses and significance: ** *p* < 0.01; *** *p* < 0.001 between the patient groups.

**Table 6:** Inflammatory and nutritional parameters in the two clusters of patients, excluding those with COPD.

	Below-Threshold Cluster	Above-Threshold Cluster
	< 4.99 × 10^3^ Cells/µL	≥ 4.99 × 10^3^ Cells/µL
	(N = 640)	(N = 275)
**Blood parameters,** $\bar{x}\;$(SD)		
Total leukocytes, cells/µL	6.19 (1.72) × 10^3^	10.61 (4.35) × 10^3^ ***
Total neutrophils, cells/µL	3.36 (0.91) × 10^3^	7.48 (2.49) × 10^3^ ***
Neutrophils, %	54.85 (9.31)	70.95 (11.1) ***
Total lymphocytes, cells/µL	2.06 (1.23) × 10^3^	2.11 (3.46) × 10^3^
Lymphocytes, %	32.64 (9.15)	19.0 (9.0) ***
Total eosinophils, cells/µL	0.20 (0.20) × 10^3^	0.19 (0.19) × 10^3^
Eosinophils, %	3.22 (2.68)	1.90 (1.88) ***
Platelets, cells/µL	240 (66) × 10^3^	284 (86) × 10^3^ ***
Alpha-1 antitrypsin, mg/dL	132.87 (35.71)	135.65 (44.16)
CRP, mg/dL	2.64 (7.72)	5.09 (9.75) ***
ESR, mm/h	16.01 (14.32)	23.2 (20.35) ***
Fibrinogen, mg/dL	394.9 (108.1)	496.1 (167.2) ***
Hemoglobin, g/dL	13.58 (1.42)	13.35 (1.65) *
Hematocrit, %	41.31 (4.02)	40.76 (4.7)
Creatinine, mg/dL	0.8 (0.43)	0.87 (0.5)
Total proteins, g/dL	7.05 (0.58)	6.97 (0.67)
Albumin, g/dL	4.25 (0.39)	4.14 (0.48) *

Continuous variables are presented as mean (standard deviation) for the study group. Definition of abbreviations: N, number; CRP, C-reactive protein; ESR, erythrocyte sedimentation rate; uL, microliter; dL, deciliter; mg, milligrams; mm, millimeters; h, hour; g, grams. Statistical analyses and significance: * *p* < 0.05; *** *p* < 0.001 between the patient groups.

**Table 7:** Inflammatory and nutritional parameters in the two clusters of patients, excluding those with chronic colonization by PA.

	Below-Threshold Cluster	Above-Threshold Cluster
	< 4.99 × 10^3^ Cells/µL	≥ 4.99 × 10^3^ Cells/µL
	(N = 547)	(N = 224)
**Blood parameters,** $\bar{x}\;$(SD)		
Total leukocytes, cells/µL	6.29 (2.87) × 10^3^	10.78 (4.68) × 10^3^ ***
Total neutrophils, cells/µL	3.33 (0.94) × 10^3^	7.56 (2.52) × 10^3^ ***
Neutrophils, %	54.08 (9.69)	70.7 (11.98) ***
Total lymphocytes, cells/µL	2.18 (2.53)	2.21 (3.82) × 10^3^
Lymphocytes, %	33.15 (9.69)	19.26 (9.64) ***
Total eosinophils, cells/µL	0.21 (0.21)	0.17 (0.18) × 10^3^ *
Eosinophils, %	3.29 (2.88)	1.67 (1.7) ***
Platelets, cells/µL	238 (67)	280 (84) × 10^3^ ***
Alpha-1 antitrypsin, mg/dL	132.64 (36.22)	137.98 (44.09)
CRP, mg/dL	2.32 (7.09)	4.66 (9.84) **
ESR, mm/h	15.07 (13.86)	20.03 (17.65) **
Fibrinogen, mg/dL	398.1 (106.2)	505.7 (160.1) ***
Hemoglobin, g/dL	13.69 (1.4)	13.6 (1.68)
Hematocrit, %	41.5 (4.05)	41.47 (4.9)
Creatinine, mg/dL	0.8 (0.39)	0.85 (0.29)
Total proteins, g/dL	7.02 (0.57)	6.86 (0.64) *
Albumin, g/dL	4.23 (0.42)	4.13 (0.46) *

Continuous variables are presented as mean (standard deviation) for the study group. Definition of abbreviations: N, number; CRP, C-reactive protein; ESR, erythrocyte sedimentation rate; uL, microliter; dL, deciliter; mg, milligrams; mm, millimeters; h, hour; g, grams. Statistical analyses and significance: * *p* < 0.05; ** *p* < 0.01; *** *p* < 0.001 between the patient groups.

## Data Availability

The datasets generated and analyzed during the current study are available from the corresponding author on reasonable request.
